# Target Achievements of Low-Density Lipoprotein Cholesterol, Blood Pressure, and Glucose in Patients with Diabetes after Acute Coronary Syndrome: Findings from the Chinese Cardiovascular Association Database – iHeart Project

**DOI:** 10.5334/gh.1400

**Published:** 2025-02-17

**Authors:** Jing Yang, Rui Zhang, Bing Han, Hui Li, Jingfeng Wang, Yihui Xiao, Xiaofan Yu, Shaofeng Guan, Cuilian Dai, Hua Yan, Tingbo Jiang, Hanbin Cui, Shuang Yang, Zeqi Zheng, Yugang Dong, Annai Wang, Guohai Su, Yan Wang

**Affiliations:** 1 Department of Cardiology, Shanghai Xuhui Central Hospital, Zhongshan-Xuhui Hospital, Fudan University, Shanghai, 200237, China; 2 China Heart House, Suzhou, 215124, China; 3 Department of Cardiology, Xuzhou Central Hospital, The Affiliated Xuzhou Hospital of Medical School of Southeast University, Xuzhou, Jiangsu, 221009, China; 4 Department of Cardiology, the second affiliated hospital of Soochow University, Suzhou, 215004, China; 5 Department of Cardiovascular Medicine, Sun Yat-sen Memorial Hospital, Sun Yat-sen University, Guangzhou, 510120, China; 6 Department of Cardiovascular Medicine, The First Affiliated Hospital of Xi’an Jiaotong University, Xi’an, 710061, China; 7 Department of Cardiology, The First Affiliated Hospital of University of Science and Technology of China, Hefei, 230001, China; 8 Department of Cardiology, Huadong Hospital, Fudan University, Shanghai, 200040, China; 9 Xiamen Cardiovascular Hospital, School of Medicine, Xiamen University, Fujian, 361000, China; 10 Department of Cardiology, Wuhan Asia Heart Hospital, Wuhan, 430022, China; 11 Department of Cardiology, The First Affiliated Hospital of Soochow University, Suzhou, 215006, China; 12 Cardiology Center, The First Affiliated Hospital of Ningbo University, Ningbo, 315000, China; 13 Department of Cardiology, The 2nd Affiliated Hospital of Harbin Medical University, Harbin, 150086, China; 14 Department of Cardiology, the first affiliated hospital of Nanchang University, Nanchang, 330006, China; 15 Department of Cardiology, the first affiliated hospital of Sun Yat-sen University, Guangzhou, 510080, China; 16 NHC key Laboratory of assisted Circulation, Sun Yat-sen University, Guangzhou, 510080, China; 17 Department of Cardiology, Jinan Central Hospital, Shandong First Medical University, Shandong, 250013, China; 18 Department of Cardiology, Xiamen Cardiovascular Hospital Xiamen University, NO. 2999 Jinshan Road, Xiamen, 200080, China

**Keywords:** Acute coronary syndrome, Diabetes, Multifactorial control, Secondary prevention

## Abstract

**Aim:**

To evaluate the achievement of metabolic risk factor targets and influencing factors in ACS patients with diabetes during the 12 months after discharge.

**Methods:**

We retrospectively analyzed data from the Chinese Cardiovascular Association database-iHeart Project. Patients who were hospitalized with a diagnosis of ACS between 2014 and 2021 and who had at least one measurement record of LDL-C, BP, or HbA_1c_ within 12 months after discharge were included. We further stratified patients by diabetes status and analyzed the correlation between clinical characteristics, measurement strategy, and achievement of targets.

**Results:**

Diabetes was identified in 1,027 (27.5%) of the eligible patients. The proportions of patients with diabetes achieving targets of LDL-C, BP, and HbA_1c_ levels were 42.4%, 61.5%, and 43.7%, respectively. However, combined achievement rate was significantly lower in patients with diabetes than patients without diabetes (16.6% vs. 26.6%). Patients with diabetes who underwent the first measurement within three months or had ≥3 measurements within 12 months were positively associated with achieving combined targets.

**Conclusions:**

The achievement of multifactorial targets among patients with ACS is suboptimal, particularly among patients with concomitant diabetes. The optimal measurement strategy post-discharge is essential for improving the comprehensive management of metabolic risk factors in ACS patients.

## 1. Introduction

Despite advances in the treatment and prevention, acute coronary syndrome (ACS) remains the leading cause of morbidity and mortality worldwide (1,2). One quarter to one third of ACS patients have known diabetes mellitus (3,4,5). The concomitant diabetes was associated with an increased risk of ACS, both in the short-term and long-term (6,7). Accumulation of metabolic risk factors, including hypertension and dyslipidemia, beyond hyperglycemia, was associated with an increased risk of major adverse cardiovascular events (MACE) (8). Therefore, in addition to lifestyle modification, multifaceted pharmacologic interventions with a patient-centered approach for control of blood pressure, glucose and atherogenic lipoproteins are the cornerstones of secondary prevention (9,10).

Treatment targets of glycated hemoglobin (HbA_1c_), blood pressure (BP), and low-density lipoprotein cholesterol (LDL-C) for secondary prevention were recommended by the practical guidelines (11). However, the achievement rates of BP, glucose, and LDL-C were suboptimal (12), with LDL-C having the lowest rate (13,14,15). The combined management of three risk factors was particularly unfavorable in patients with concomitant diabetes (16), which confers considerable residual CV risk (17). Adequate monitoring reflecting patient adherence and physician engagement are critical to control of risk factors. However, monitoring strategy recommended by guidelines is not routinely followed in clinical practice. A recent real-world study demonstrated only 52% of patients who underwent a percutaneous coronary intervention (PCI) had an LDL-C measurement within six months after the procedure (18). Currently, the majority of research on achieving comprehensive targets in diabetes primarily focuses on primary prevention (19,20,21,22). However, investigation on multifactorial measurements and target achievements in a large-scale population receiving contemporary therapies for ACS and concomitant diabetes is still limited.

Using a multi-center database derived from electronic health records (EHRs), we investigated target achievement of metabolic risk factors in patients with diabetes after hospitalization for ACS. Moreover, we analyzed the impact of clinical characteristics and follow-up strategies on achieving multifactorial control.

## 2. Methods

### 2.1 Study design and data source

Our study retrospectively examined data from the Chinese Cardiovascular Association (CCA) Database-iHeart Project, which was designed to establish a nationwide platform utilizing EHRs for real-world evidence. Twenty tertiary hospitals collaborated in this project, collecting clinical data including medical history, physical examinations, comorbid conditions, laboratory tests, imaging reports, medication prescriptions, and operation information from eligible patients admitted to the cardiology department between January 1, 2014, and December 31, 2021. The data were extracted and structured using natural language processing techniques, incorporating standardized terminologies and codes to ensure seamless interoperability among participating hospitals. Quality control measures were implemented for data sharing and subsequent analysis, with the CCA steering committee overseeing standards and proposals to maintain research integrity. Patient data confidentiality is prioritized, with all data identified and analyzed under a protocol that emphasizes privacy protection. The applications for research purposes must undergo a comprehensive review and obtain approval from the academic committee. Researchers are obligated to strictly adhere to established protocols and regulations in order to ensure robust security measures when accessing patient data.

### 2.2 Study population

**Figure 1:**
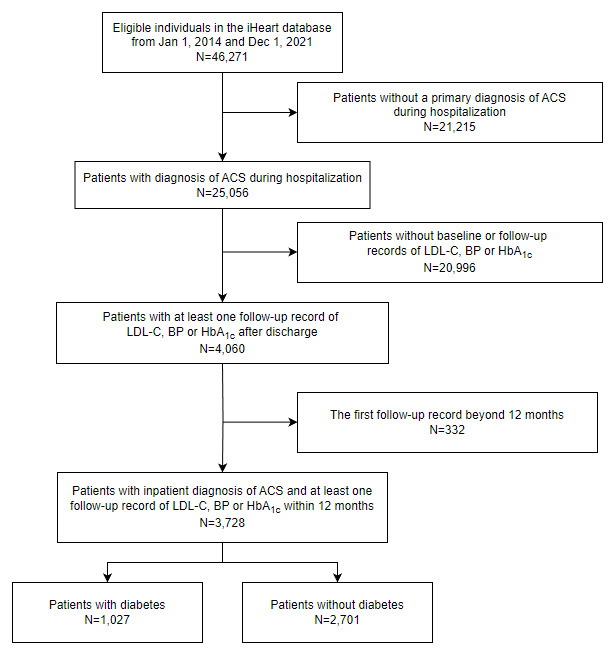
Flowchart of patient selection.

In the present study, patients hospitalized with a primary diagnosis of ACS between January 1, 2014, and December 31, 2021, were enrolled. Eligible patients were identified through discharge records corresponding to the international classification of diseases, Tenth Revision codes I20.0, I20.1, I21-I21.9, I22, and I23. Exclusion criteria entailed the absence of baseline or 12-month follow-up records for measurements of blood glucose, lipid profiles, or BP, as delineated in Figure 1. The enrolled patients were further stratified according to the presence or absence of diabetes. Ethical approval was provided by the Ethics Committee of Shanghai Xuhui Central Hospital (Approval Number: 2023-014). Given the retrospective and anonymous nature of data collection, devoid of unique patient identifiers, written informed consent was deemed unnecessary for this analysis.

### 2.3 Clinical covariates

Baseline characteristics of the patients were obtained from EHR databases of index hospitalization for ACS. Demographic covariates and vital signs were obtained from admission records. Comorbidities were ascertained from medical history and discharge records. Definitions of clinical covariates, including dyslipidemia, hypertension, diabetes, smoking, atrial fibrillation, prior myocardial infarction (MI), and cerebrovascular disease, are provided in **Table S1**. Laboratory tests were defined as the initial measurement after admission, encompassing fasting glucose (FG), fasting lipid panel, estimated glomerular filtration rate (eGFR), D-dimer, neutrophil percentage (NEU%), creatinine (Cr) and N-terminal pro-brain natriuretic peptide (NT-proBNP). The implementation of invasive therapies, including PCI and coronary artery bypass graft (CABG) during hospitalization, was extracted and confirmed based on the discharge diagnosis code, procedure report, and physician order. Medication information was obtained from the discharge record. For categorical data, the presence and absence of a diagnostic code/medication prescription in the EHR were coded as “1” and “0” respectively. Continuous features and patients with >60% missing values were discarded, and the remaining values were imputed using random forest-based algorithms.

### 2.4 Follow-up and outcome definition

Patients with ACS were advised to participate in a comprehensive cardiac rehabilitation program prior to discharge, which includes measures for secondary prevention. In accordance with clinical practice guidelines, a standardized protocol had been implemented for the post-discharge follow-up of patients with ACS. The follow-up plan was established by the attending cardiologists and adjusted as necessary. Upon discharge from the hospital, patients were advised to undergo regular follow-up evaluations at 1 month, 3 months, 6 months, and 12 months. Patient follow-up was typically conducted by attending cardiologists or by dedicated follow-up teams comprising cardiologists, nurses, and clinical fellows of cardiology department. Follow-up visits were typically conducted during outpatient services. A follow-up measurement was defined as any documented testing for LDL-C, BP, or HbA_1c_ conducted after the discharge. In accordance with the local guidelines on secondary prevention for ACS, the primary outcome was the achievement of multifactoral targets, defined as BP <140/90 mmHg, LDL-C <1.8 mmol/L, HbA_1c_ <7.0% (if diabetes), based on the latest outpatient laboratory results within a 12-month period following discharge.

### 2.5 Statistical analysis

Descriptive statistics were presented as mean ± SD, median (Q1–Q3), or percentage. To compare groups, we used the Mann-Whitney test for continuous variables and the Chi-square (χ^2^) test for dichotomous variables. Logistic regression analysis calculating the odds ratio (OR) with a 95% confidence interval (CI) was used to analyze the factors influencing achieving target LDL-C, BP, and HbA_1c_. For logistic regression models, we performed stepwise adjustment as follows: Model 1 = unadjusted; Model 2 = adjustment for ACS subtype, lipid-lowering medication, and hypertension; Model 3 = adjustment for Model 2 + total cholesterol (TC), high-density lipoprotein cholesterol (HDL-C), FG, Cr, NEU%, D-dimer and log (NT-proBNP). A *P*-value of < 0.05 is considered statistically significant. All the analyses were performed using R Statistical Software, version 4.1.2.

## 3. Results

### 3.1 Baseline characteristics of patients with ACS

The mean age (SD) was 73.3 (12.5) years, with 2,395 males (64.2%). Diabetes was present in 1,027 patients (27.5%) with a median HbA_1c_ of 7.4% (6.5%, 8.7%). Patients with diabetes were older and had a higher proportion of female, NSTEMI, invasive coronary intervention, and cerebrovascular disease than those without diabetes. The lipid profile was similar between patients with or without diabetes. Further characteristics are detailed in Table 1.

**Table 1:** Baseline characteristics of study patients.


PATIENT CHARACTERISTICS	ALL	DIABETES	NON-DIABETES	*p* VALUE

	(n = 3728)	(n = 1027)	(n = 2701)	

Age, yr	73.3 (12.5)	74.8 (11.3)	72.7 (12.9)	<0.001

Sex				<0.001

Male	2395 (64.2%)	586 (57.1%)	1809 (67.0%)	

Female	1333 (35.8%)	441 (42.9%)	892 (33.0%)	

**Index hospitalization for ACS**				

ACS subtype				

NSTEMI	523 (14.0%)	180 (17.5%)	343 (12.7%)	<0.001

STEMI	1164 (31.2%)	232 (22.6%)	932 (34.5%)	

UA	1790 (48.0%)	528 (51.4%)	1262 (46.7%)	

Undetermined	251 (6.7%)	87 (8.5%)	164 (6.1%)	

SBP, mmHg	136 (21)	140 (22)	135 (20)	<0.001

DBP, mmHg	79 (12)	78 (12)	79 (12)	0.655

PCI or CABG	1650 (44.3%)	503 (49.0%)	1147 (42.5%)	<0.001

**Laboratory values**				

FG, mmol/L	6.7 (5.3–9.0)	8.6 (6.5–11.9)	6.2 (5.1–7.9)	<0.001

HbA_1c_, %	6.2 (5.7–7.1)	7.4 (6.5–8.7)	6.0 (5.6–6.4)	<0.001

TC, mmol/L	4.5 (3.8–5.4)	4.5 (3.7–5.4)	4.5 (3.8–5.4)	0.249

TG, mmol/L	1.4 (1.0–2.0)	1.5 (1.1–2.3)	1.4 (1.0–2.0)	<0.001

LDL-C, mmol/L	2.8 (2.2–3.4)	2.7 (2.1–3.5)	2.8 (2.2–3.4)	0.315

HDL-C, mmol/L	1.1 (0.9–1.3)	1.0 (0.9–1.3)	1.1 (0.9–1.3)	<0.001

Non-HDL-C, mmol/L	3.5 (2.9–4.0)	3.5 (2.9–4.0)	3.5 (2.9–4.0)	0.833

Lp (a), mg/L	263 (123–400)	253 (110–417)	267 (128–394)	0.408

NT-proBNP, pg/ml	688 (306–1856)	711 (314–1977)	673 (304–1766)	0.216

NEU, %	70.6 (61.3–80.0)	68.8 (60.4–78.0)	71.8 (61.6–80.7)	<0.001

eGFR, mL/min/1.73 m^2^	84.4 (66.8–98.6)	85.1 (63.1–99.3)	84.3 (67.7–98.2)	0.575

Cr, μmol/L	80 (66–100)	78 (63–105)	81 (67–98)	0.232

D-dimer, mg/L	0.5 (0.3–1.0)	0.5 (0.3–1.0)	0.5 (0.3–1.0)	0.554

**Comorbidities**				

Dyslipidemia	2694(72.3%)	778 (75.8%)	1916 (71.0%)	0.004

Hypertension	2056(55.2%)	792 (77.1%)	1264 (46.8%)	<0.001

Smoker	1333(35.8%)	405 (39.4%)	928 (34.4%)	<0.001

Atrial fibrillation	257(6.9%)	79 (7.7%)	178 (6.6%)	0.340

Prior MI	895(24.0%)	349 (34.0%)	546 (20.2%)	<0.001

Cerebrovascular disease	740(19.8%)	310 (30.2%)	430 (15.9%)	<0.001


Abbreviations: ACS, acute coronary syndrome; NSTEMI, non-ST-elevation myocardial infarction; STEMI, ST-segment elevation myocardial infarction; UA, unstable angina; SBP, systolic blood pressure; DBP, diastolic blood pressure; PCI, percutaneous coronary intervention; CABG, coronary artery bypass grafting; FG, fasting glucose; HbA_1c_, glycated hemoglobin; TC, total cholesterol; TG, triglyceride; LDL-C, low-density lipoprotein cholesterol; HDL-C, high-density lipoprotein cholesterol; Lp (a), lipoprotein (a); NT-proBNP, N-Terminal pro-brain natriuretic peptide; eGFR, estimated glomerular filtration rate; Cr, creatinine; MI, myocardial infarction. Data are n (%), mean (SD), or median (IQR). *P* values for comparison between diabetes and non-diabetes.

Among patients with diabetes, 86.8% received glucose-lowering medication, 98.3% received lipid-lowering medication, and 93.4% received antihypertensive medication, significantly higher than in patients without diabetes (P < 0.01). The proportion of diabetes patients treated with all three classes of therapies was 82.2%. Additional detail is provided in **Table S2**.

### 3.2 Measurements and target achievements after discharge within 12 months

Measurement records of ≥ 3, 2, and 1 within 12 months of discharge were identified in 1,401; 761; and 1,566 patients, respectively. Within three months after discharge, 2,265 (60.8%) patients underwent the first measurement. The median time to the first measurement from discharge was 27.0 days.

**Figure 2:**
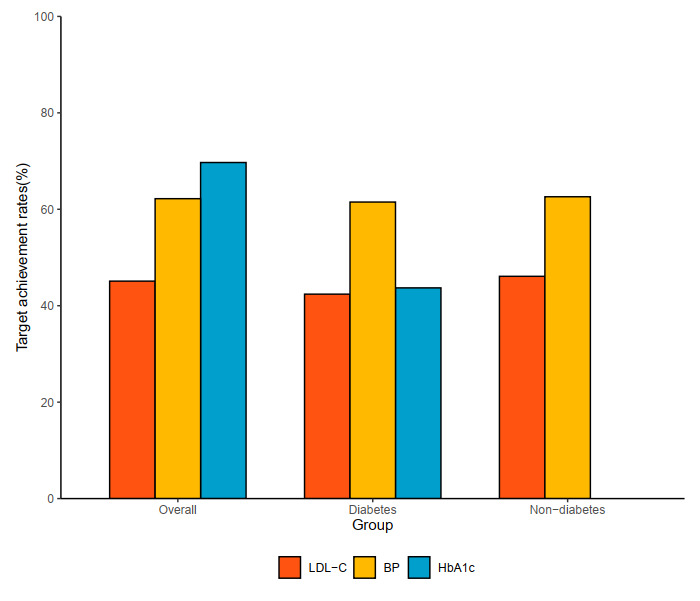
Target Achievement Rate of LDL-C, BP and HbA_1c_.

Overall, proportions of patients achieving targets of LDL-C, BP and HbA_1c_ were 45.1%, 62.2%, and 69.7%, respectively. Among patients with diabetes, the target achievement rates were 42.4%, 61.5%, and 43.7%, respectively. Only 16.6% of patients with diabetes achieved control targets for all three risk factors. The achievement of control targets is shown in Figure 2.

### 3.3 Clinical predictors and measurement records of target achievement

For individual target achievement, after fully adjusted, patients with MI (adjusted OR, 0.411[95% CI 0.217–0.778) and atrial fibrillation (adjusted OR, 0.384[95% CI 0.152–0.973) were less likely to achieve targets of HbA_1c_. In the fully adjusted model, patients who had ≥3 measurements were more likely to achieve the LDL-C target (adjusted OR, 2.392[95% CI 1.264–4.524; P = 0.007), compared with those undergoing <3 measurements. The first measurement occurring within three months of discharge was positively associated with LDL-C achievement (adjusted OR, 2.091[95% CI 1.095–3.993; P = 0.025). Other factors associated with individual target achievement are detailed in Table 2.

**Table 2:** Association of clinical variables and achievement of LDL-C, BP and HbA_1c_ in diabetes patients.


VARIABLE	LDL-C	BP	HBA_1C_

	OR (95% CI)	OR (95% CI)	OR (95% CI)

Age >65 yr	0.508(0.212–1.218)	0.861(0.387–1.914)	0.540(0.236–1.233)

Male	1.471(0.763–2.836)	1.084(0.580–2.024)	1.257(0.658–2.401)

Revascularization	1.309(0.682–2.510)	1.216(0.655–2.257)	1.185(0.627–2.238)

Prior MI	0.704(0.366–1.357)	1.003(0.541–1.859)	0.411(0.217–0.778)*

Atrial fibrillation	1.146(0.435–3.024)	1.633(0.641–4.162)	0.384(0.152–0.973)*

eGFR <60 mL/min/1.73 m²	1.113(0.465–2.662)	0.446(0.198–1.004)	2.286(0.984–5.313)

Newly diagnosed MI	2.403(0.931–6.207)	1.847(0.772–4.418)	1.003(0.415–2.422)

Measurements ≥3 times	2.392(1.264–4.524)	1.420(0.788–2.560)	1.829(0.997–3.356)

First measurement ≤3 months	2.091(1.095–3.993)	1.679(0.904–3.118)	0.998(0.531–1.875)


Abbreviations: OR, odds ratio; MI, myocardial infarction; eGFR, estimated glomerular filtration rate; *P < 0.05. ORs (95% CI) of achievement of LDL-C, BP, and HbA_1c_ were fully adjusted models.

**Figure 3:**
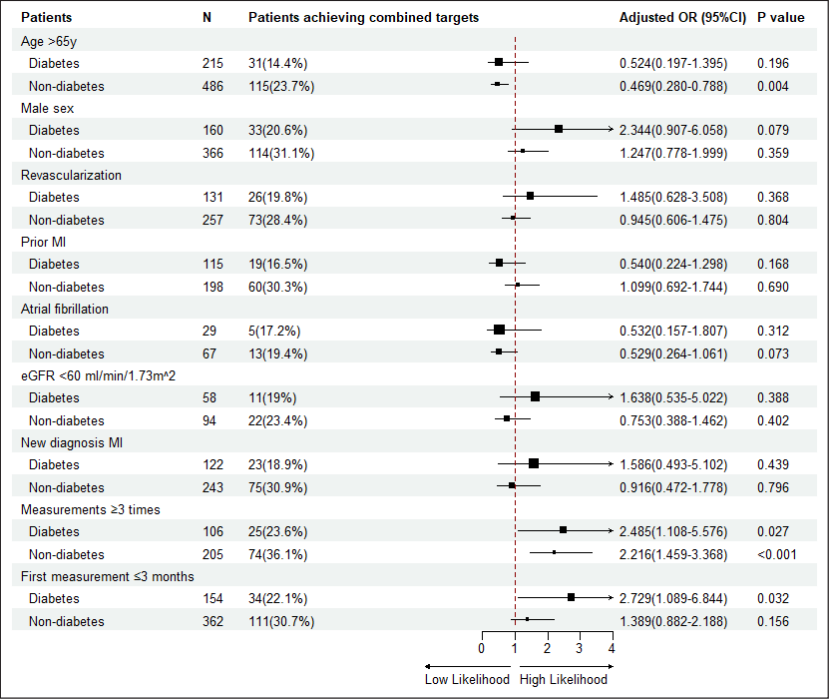
Multivariate logistic analysis of risk for combined achievements in patients with or without diabetes.

After full adjustment, no significant association was observed between clinical variables and combined target achievement in patients with diabetes. In contrast, for patients without diabetes, age >65 was inversely associated with the achievement of combined target achievement. Patients who had ≥3 measurements were more likely to achieve the combined target achievement irrespective of diabetes status. Patients with diabetes who had the first measurement within three months of discharge were positively associated with LDL-C achievement (adjusted OR, 2.729[95% CI 1.089–6.844; P = 0.032) (Figure 3). Results of univariate and multivariable analysis for subgroups stratified by diabetes diagnosis are shown in **Table S3**.

## 4. Discussion

The main findings of the present study were summarized as follows: (1) Among individuals with diabetes, the rates of target achievement for LDL-C, BP, and HbA_1c_ were 42.4%, 61.5%, and 43.7%, respectively; (2) Only 16.6% of patients with diabetes achieved control targets for all three risk factors; (3) No association between the clinical variables and combined target achievement was observed in patients with diabetes, while age >65 was inversely associated with combined target achievement in patients without diabetes; and (4) Patients who had undergone three or more measurements within 12 months after discharge were more likely to achieve the target, in both patients with or without diabetes. A positive association was observed in patients with diabetes with combined targets when the first measurement occurred within three months of discharge.

### 4.1 Multifactorial target achievement

Optimal control of CVD risk factors is one of the most effective approaches for secondary prevention in patients with ACS. Findings from the current study were consistent with previous studies. Although the proportion of people achieving targets varied considerably between studies, suboptimal control of risk factors remains a global challenge, especially in patients with diabetes (23). Despite the substantial disparity between the available evidence and real-world scenarios, this gap may be widening as a result of the progressively lower LDL-C targets (24). The large gap between the low target achievement rate and the high prescription of medical treatment for secondary prevention in our study provided insight into potential mechanisms of patient adherence and physician inertia.

Firstly, medication adherence plays a crucial role in the target achievement of risk factors. Patients need to take medications on time with the prescribed dosage and undergo adequate monitoring to ensure safety and efficacy in accordance with the physician’s advice. However, achieving more targets increases the pill burden and risk for adverse effects, thereby decreases adherence. Secondly, lack of awareness and knowledge of physicians is also a contributing factor. Physicians need to be familiar with the best medication regimens, provide individualized treatment based on the specific conditions of each patient, and make timely medication adjustments. However, in daily practice, physicians may not give sufficient attention to and promote the implementation of these measures.

Not all risk factors are equally important in reducing CVD risk. As shown in a recent study, in patients with diabetes, achieving target of LDL-C was associated with the most significant reduction of CVD risk among three risk factors, suggesting that LDL-C control should be prioritized in attaining combined targets (25). However, similar to previous studies, LDL-C with the lowest achievement rate was identified among the three risk factors with background treatment of statins (16,26,27). In addition to the unawareness of the priority of LDL-C control, the lack of a standard framework for effective monitoring and management may not be neglected. Furthermore, unlike glucose and BP, which can be easily tested or monitored at home using non-invasive or minimally invasive modalities, LDL-C measurement requires venous blood sampling, which can solely be conducted within healthcare facilities. Consequently, limited accessibility might discourage individuals from regularly tracking their LDL-C levels and thus contribute to the lowest achievement rate observed in our study.

Patients with diabetes are critical to focus on for being at substantially elevated CVD risk (17). Some studies indicated that the achievement rates among patients with diabetes are significantly lower than patients without diabetes (28). The results of the current study demonstrated comparable achievement rates between patients with or without diabetes for individual indicators and lower achievement rates in patients with diabetes for combined target. In line with previous studies in patients with concomitant diabetes for secondary prevention (16,29) or primary prevention (30,32,33), combined target achievement in the current study was worse than individual target control. In real-world practice, the management of diabetes, hypertension, and dyslipidemia is conducted by different disciplines. Inconsistent perspectives and strategies delivered by fragmented healthcare pose challenges to the management of CVD in patients with concomitant diabetes.

### 4.2 Characteristics and measurements of target achiever

Identifying characteristics associated with risk factor control can help develop a more tailored secondary prevention strategy. Regarding LDL-C, the pivotal target, multivariable analysis in the current study showed that patients with MI or atrial fibrillation were less likely to achieve targets. This implies that individuals with ACS and comorbidities should prioritize monitoring their lipid levels more attentively. Follow-up is essential for chronic management post-discharge. The 2018 ACC/AHA cholesterol guidelines (34) and the recent ESC guidelines (9) recommend obtaining a lipid panel about four weeks after each lipid-lowering therapy is initiated or adjusted to determine whether treatment targets have been achieved. Several recent studies demonstrated that monitoring lipid panels during the follow-up period may improve statin adherence (35,36). Intensification of lipid-lowering therapy was observed with the increased number of lipid panels in a dose-dependent pattern (37,38). Measurement may improve patient adherence via awareness of numerical changes, monitoring the response and safety, and enabling personalized medication. However, it is important to note that the specific guidelines governing the frequency and type of these follow-up engagements are not rigorously defined, a situation that can be attributed to the ambiguous nature of the guidelines and the variability in patient compliance. Furthermore, multifactorial control is more complex than the management of an individual risk factor. Currently, available guidelines only recommend control targets of metabolic risk factors without detailed instructions on the management strategy for a combination of multiple risk factors (41).

The current study extends previous findings by providing evidence of evolving optimal number (≥3 within 12 months) and timing (initiated within three months) of measurement. We found that patients who had ≥3 measurements were more likely to achieve the combined targets with both diabetes and non-diabetes groups. For patients with diabetes, it is advisable to undergo the first measurement within three months of discharge for improving target achievement. Furthermore, these findings also imply the rationale for early initiation of follow-up or re-examination in patients with diabetes with ACS after discharge. Notably, measurement in the current study was defined as any lipid, BP, or glucose test. Even without a lipid panel, patients undergoing measurement of glucose or BP were more likely to control LDL-C. A recent study (31) indicated that failure to achieve one target was associated with failure to achieve control of other risk factors. Our findings provide further understanding to support the hypothesis of mutual influences in diverse risk factor control.

### 4.3 Target of metabolic risk factor control

Multifactorial management simultaneously confers great benefits to reducing MACEs. In clinical practice and our study, guideline-recommended targets were adopted. The current guidelines advocate for a multifaceted approach to management (21). However, the extent of combined targets remains unclear. Results from recent studies do not fully support the efficacy of intensified multifactorial intervention compared to standard care for the primary prevention of a composite of MACEs (21,22). A similar reduction in percent atheroma volume was observed when achieving a lenient target (LDL-C <100 mg/dL and BP <140/90 mmHg) compared with an aggressive target (LDL-C <70 mg/dL and BP <120/70 mmHg) (39). The synergistic effect derived from multifactorial treatment suggests that intensive treatment on top of lenient targets may not further enhance benefit. Therefore, head-to-head research is required to determine the optimal level for combined control regarding the effectiveness of different strategies in patients at diverse risk.

### 4.4 Clinical implication and perspective

The results have several important implications. First, the findings support the potential of an appropriate measurement strategy for multifactorial management of ACS patients with diabetes because of their low achievement rates of combined targets. The number and timing of measurements which facilitated multifactorial target achievement in the current study are hypothesis-generating rather than conclusive, which requires further study to determine the optimal measurement strategy. In addition, innovation in healthcare delivery may address issues related to patient adherence, physician inertia, and accessibility to medical resources. A recent study (40) demonstrated the implementation of a coordinated, multifaceted intervention, including identifying local prescription barriers, developing the interdisciplinary framework, coordinating physician collaboration, providing education to physicians and patients, real-time reporting to care providers, and increasing the prescription of evidence-based therapies in patients with diabetes and ASCVD. In this context, a novel healthcare model involving a dedicated clinic comprising individualized targeting of risk factors, guidance on lifestyle modifications, and an algorithm for medication adjustment based on a structured, multidisciplinary network may provide patients at high risk of CVD with comprehensive evaluation and cardiovascular risk reduction.

### 4.5 Limitations

Several limitations in the current study should be considered. First, patients with diabetes were identified through discharge diagnosis in this study. Complete data of glucose diagnostic tests such as oral glucose tolerance tests and random plasma glucose were unavailable, potentially leading to missed cases of diabetes. However, criteria for diabetes testing differs widely across regions, and a comprehensive screening programme is yet to be developed. Second, longitudinal data, including clinical outcomes, would have strengthened our findings. Nevertheless, failure to achieve guideline-recommended targets signifies an elevated risk for subsequent cardiovascular events. Therefore, the utilization of achievement rates as surrogate endpoints emerges as a prudent choice. Third, the specific targets in this study were determined based on the current national guidelines, which differ from other international guidelines. Consequently, these disparities have also contributed to variations in the results. However, there is currently no consensus among international guidelines on the optimal targets. Fourth, our study only comprised Chinese patients. Thus, our conclusions may not be generalizable to other ethnicities. However, the consistency of suboptimal achievement of risk factors with previous studies reflects the ubiquity of the challenge worldwide and potential avenues for improvement in terms of low adherence and suboptimal care delivery. Last, The retrospective design of this study possesses inherent limitations that compromise the robustness of the research findings. However, a similar conclusion was reached in the sensitivity analysis. A further randomized clinical trial would be required to reassess our results.

## 5. Conclusions

Multifactorial target achievements within 12 months after discharge are suboptimal for ACS patients with concomitant diabetes. Appropriate measurement timing and frequency during follow-up may facilitate target achievement. Incorporating a predetermined measurement strategy into a standard follow-up plan and establishing a multidisciplinary collaboration framework may improve the management of metabolic risk factors and cardiovascular prognosis.

## Data Accessibility Statement

The data that support the findings of this study are collected from iHeart Project of Chinese Cardiovascular Association, but restrictions apply to the availability of these data, which were used under license for the current study, so they are not publicly available. Data are, however, available from the authors upon reasonable request and with permission from iHeart Program of Chinese Cardiovascular Association.

## Additional File

The additional file for this article can be found as follows:

10.5334/gh.1400.s1 Supplementary Material: Tables S1 to S3. Definition of clinical covariates.

## Figures and Tables

**Table:** 

ACS	Acute coronary syndrome
LDL-C	Low-density lipoprotein cholesterol
SBP	Systolic blood pressure
HbA_1c_	Glycated hemoglobin
CVD	Cardiovascular disease
MACE	Major adverse cardiovascular events
BP	Blood pressure
EHRs	Electronic health records
CCA	Chinese Cardiovascular Association
STEMI	ST-segment elevation myocardial infarction
NSTEMI	Non-ST-elevation myocardial infarction
UA	Unstable angina
FG	Fasting glucose
eGFR	Estimated glomerular filtration rate
Cr	Creatinine
NT-proBNP	N-Terminal pro-brain natriuretic peptide
PCI	Percutaneous coronary intervention
CABG	Coronary artery bypass graft
OR	Odds ratio
CI	Confidence interval
TC	Total cholesterol
HDL-C	High-density lipoprotein cholesterol
MI	Myocardial infarction
