# Supplementary material for: Target Achievements of Low-Density Lipoprotein Cholesterol, Blood Pressure, and Glucose in Patients with Diabetes after Acute Coronary Syndrome: Findings from the Chinese Cardiovascular Association Database – iHeart Project
Source: Glob Heart. 2025 Feb 17;20(1):14. doi: 10.5334/gh.1400 (PMC11843988; doi:10.5334/gh.1400)
Supplement: Supplementary Material: Tables S1 to S3. — Definition of clinical covariates. [file gh-20-1-1400-s1.pdf]

Supplementary Material

Table S1. Definition of clinical covariates.

Table S2. Lipid-, blood pressure-, and glucose-lowering therapies.

Table S3. Univariate and multivariate logistic analysis of factors influencing combined achievement of the LDL-C, BP and HbA_{1c} for diabetic patients and the LDL-C, BP for non-diabetic patients.

Table S1 Definition of clinical covariates

Clinical covariates	Definition
Dyslipidemia	A previous diagnosis of dyslipidemia, currently receiving any lipid-lowering drug, or total cholesterol >5.18 mmol/L, LDL-C ≥3.37 mmol/L, HDL-C <1.04 mmol/L in men and <1.30 mmol/L in women, or fasting triglycerides >1.7 mmol/L in those with concomitant lipid values.
Hypertension	Self-report, having a diagnosis of hypertension made by a healthcare professional prior to this care encounter, diagnosis by 3 consecutive office blood pressure measurements with systolic blood pressure ≥140 mmHg and or diastolic blood pressure ≥90 mmHg, or treatment of hypertension.
Diabetes	Fasting plasma glucose >7.0 mmol/L, 2-h plasma glucose ≥11.1 mmol/L during an oral glucose tolerance test, hemoglobin A_{1c} ≥6.5%, or diagnosis or treatment of diabetes
Smoker	Former smoker (a patient who quit smoking tobacco >1 month ago) plus current smoker (a patient who is currently smoking tobacco at a regular basis)
Atrial fibrillation	Atrial fibrillation is present within 2 wk before the current encounter.
Prior MI	Any MI occurrence between birth and arrival at this facility, excluding a presenting MI
Cerebrovascular disease	Ischemic stroke: confirmed neurological deficit of abrupt onset caused by blockage of a blood vessel supplying the brain that did not resolve within 24 h.<br>TIA: Loss of neurological function that was abrupt in onset but with complete return of function within 24 h.<br>Hemorrhagic stroke: confirmed neurological deficit of abrupt onset caused by bleeding into or around the brain.

Table S2 Lipid-, blood pressure-, and glucose-lowering therapies

	All (n=3728)	Diabetes (n=1026)	Non-diabetes (n=2702)	P value
Lipid-lowering therapies	3614(96.9%)	1009 (98.3%)	2605 (96.5%)	0.006
Statin	3614(96.9%)	1009 (98.3%)	2605 (96.5%)	0.006
PCSK-9 inhibitors	28(0.8%)	3 (0.3%)	25 (1.0%)	0.074
BP-lowering drugs	3374(90.5%)	959(93.4%)	2415(89.4%)	<0.001
ACEI/ARB	2659(71.3%)	801 (78.0%)	1858 (68.8%)	<0.001
Beta-blockers	3162(84.8%)	913(88.9%)	2249(83.3%)	<0.001
CCB	589(15.8%)	202 (19.7%)	387 (14.3%)	<0.001
ARNI	178(4.8%)	50 (4.9%)	128 (4.7%)	0.936
Glucose-lowering drugs	891(23.9%)	891(86.8%)	-	<0.001

ACEI/ARB, angiotensin-converting enzyme inhibitors/angiotensin II receptor blocker; CCB, calcium channel blockers; ARNI, Angiotensin receptor neprilysin inhibitor.

Table S3 Univariate and multivariate logistic analysis of factors influencing combined achievement of the LDL-C, BP and HbA_{1c} for diabetic patients and the LDL-C, BP for non-diabetic patients.

Variable	Diabetes		Non-diabetes	
	OR（95%CI）	P value	OR（95%CI）	P value
**Univariate**				
Age >65 yr	0.449(0.209-0.965)	0.040	0.482(0.314-0.740)	0.001
Male	2.313(1.084-4.933)	0.030	1.859(1.257-2.748)	0.002
Revascularization	1.617(0.830-3.150)	0.158	1.172(0.814-1.687)	0.393
Prior MI	0.990(0.512-1.913)	0.975	1.317(0.903-1.923)	0.153
Atrial fibrillation	1.053(0.378-2.932)	0.922	0.634(0.336-1.195)	0.159
eGFR <60 mL/min/1.73m²	1.236(0.580-2.636)	0.583	0.817(0.488-1.369)	0.443
Newly diagnosed MI	1.359(0.705-2.620)	0.360	1.434(0.995-2.066)	0.053
Measurements ≥3 times	2.315(1.190-4.504)	0.013	2.036(1.404-2.953)	<0.001
First measurement ≤3 months	3.022(1.382-6.607)	0.006	1.715(1.166-2.521)	0.006
**Multivariate**				
**Model 1**				
Age >65 yr	0.532(0.225-1.260)	0.152	0.602(0.378-0.961)	0.034
Male	2.355(0.998-5.556)	0.050	1.558(1.014-2.393)	0.043
Revascularization	1.234(0.566-2.689)	0.597	0.795(0.526-1.204)	0.279
Prior MI	0.552(0.246-1.239)	0.150	1.030(0.665-1.596)	0.894
Atrial fibrillation	0.707(0.235-2.124)	0.536	0.619(0.319-1.203)	0.157
eGFR <60 mL/min/1.73m²	1.211(0.502-2.919)	0.670	0.843(0.482-1.475)	0.550
Newly diagnosed MI	0.949(0.417-2.158)	0.900	1.173(0.752-1.829)	0.483
Measurements ≥3 times	2.559(1.200-5.457)	0.015	1.981(1.339-2.931)	0.001
First measurement ≤3 months	2.280(0.957-5.434)	0.063	1.400(0.920-2.131)	0.117
**Model 2**				
Age >65 yr	0.513(0.213-1.235)	0.137	0.601(0.374-0.966)	0.036
Male	2.359(0.993-5.601)	0.052	1.532(0.995-2.359)	0.053
Revascularization	1.194(0.541-2.635)	0.660	0.795(0.522-1.209)	0.283
Prior MI	0.541(0.239-1.227)	0.142	1.070(0.688-1.664)	0.764
Atrial fibrillation	0.677(0.223-2.051)	0.490	0.663(0.340-1.294)	0.229
eGFR <60 mL/min/1.73m²	1.351(0.533-3.423)	0.526	0.846(0.478-1.499)	0.567
Newly diagnosed MI	1.190(0.475-2.979)	0.710	0.960(0.546-1.687)	0.887
Measurements ≥3 times	2.529(1.177-5.437)	0.017	2.070(1.392-3.078)	<0.001
First measurement ≤3 months	2.221(0.922-5.353)	0.075	1.370(0.897-2.093)	0.145

Abbreviations: OR, odds ratio; MI, myocardial infarction; eGFR, estimated glomerular filtration rate. Model 1 = unadjusted; Model 2 = adjustment for ACS subtype, lipid-lowering medication and hypertension.
